# Three new species of *Pholiotina* (*Bolbitiaceae*, *Agaricales*) from China

**DOI:** 10.3897/mycokeys.139.201595

**Published:** 2026-08-27

**Authors:** Ji-li Deng, Han-bing Song

**Affiliations:** 1 College of Agriculture and Biological Science, Dali University, Dali 671003, Yunnan, China College of Agriculture and Biological Science, Dali University Dali China; 2 College of Plant Protection, Jilin Agricultural University, Changchun 130118, China College of Plant Protection, Jilin Agricultural University Changchun China

**Keywords:** Infrageneric division, morphology, new taxa, phylogenetic placement

## Abstract

In this study, maximum likelihood and Bayesian inference phylogenetic trees were reconstructed for *Pholiotina* based on the phylogenetic framework of the genus established by Song and Bau and on a concatenated dataset of internal transcribed spacer (ITS), nuclear large subunit ribosomal DNA (28S), and translation elongation factor 1-alpha (*tef*1-α) sequences. The phylogenetic trees resolved *Pholiotina* into two monophyletic clades: one containing sect. *Pholiotina* and the other comprising sect. *Vestitae* together with sect. *Intermediae*. The infrageneric subdivision of the genus was also reviewed, and the taxonomic issues that remain to be addressed were proposed. By further integrating morphological evidence, three new species of *Pholiotina* were discovered from Jilin Province and the Xizang Autonomous Region: *P. constrictibasidiata* (characterized by basidia with a median constriction), *P. raribasidiata* (notable for its rare basidia and basidiospores), and *P. jilinensis* (distinguished by the absence of pileocystidia and germ pores, with its type locality in Jilin City). In addition, this study provides an identification key to the new species, together with morphological descriptions and line drawings.

## Introduction

*Pholiotina* Fayod (*Basidiomycota*, *Agaricomycetes*, *Agaricales*, *Bolbitiaceae*) is a genus of small agaricoid fungi distributed worldwide (Hausknecht 2009; Song 2025). Members of this genus are predominantly saprotrophic, occurring in grasslands, sandy soils, decaying wood, and humus layers. To date, psilocybin has been detected in only a few species, and other chemical constituents remain largely unexplored, warranting further investigation (Hallen et al. 2003; Wu et al. 2019). Originally, the group was placed in tribe *Galera* until Fayod (1889) erected the genus *Pholiotina* and defined it as follows: pileus often with a fibrillose veil, sometimes viscid, and a hymeniform pileipellis; stipe annulate; hymenial cells long-clavate; basidia 2- or 4-sterigmate; subhymenium and hymenial base densely arranged; lamellar trama distinct; basidiospores rusty brown, ovoid to ellipsoid, slightly pointed at the apex, with a distinct germ pore; cystidia thin-walled, cylindrical (Fries 1821; Fayod 1889).

The taxonomic history of *Pholiotina* was recently reviewed in chronological detail by Song and Bau (2024) and Song (2025). These studies resolved the polyphyly of *Pholiotina* s.l. and, based on morphological and phylogenetic evidence, divided it into three genera: *Pholiotina*, *Conocybula* T. Bau & H.B. Song, and *Conobolbitina* T. Bau & H.B. Song. They also revised the circumscription of *Pholiotina*, restricting the genus to those taxa having a well-developed veil, and recognized only three sections within it: sect. *Pholiotina*, sect. *Vestitae* (Watling) Hauskn. & Krisai, and sect. *Intermediae* (Watling) Singer.

Section *Pholiotina* is mainly characterized by a distinctly annulate stipe and non-lecythiform cheilocystidia. Within the traditional classification framework of Hausknecht and Krisai-Greilhuber (2007), the section is divided into three series: ser. *Aporos* Hauskn. & Krisai, in which the basidiospores lack a germ pore; ser. *Vexans* Hauskn. & Krisai, with basidiospores that have a germ pore and lageniform cheilocystidia; and ser. *Teneroides* Hauskn. & Krisai, which also has a germ pore but includes some utriform cheilocystidia. These three series are distinguished mainly by whether the basidiospores have a germ pore and by the shape of the cheilocystidia. As of 2026, approximately 28 species (including varieties) of this section are known worldwide, whereas only seven have been reliably recorded from China, all from the northeastern region (Hausknecht 2009; Karich 2020; Song 2025). Given China’s vast territory and ecological diversity, additional species of this section are likely to be found in other parts of the country.

Section *Vestitae* is mainly characterized by the presence of veil remnants on the pileus and by cheilocystidia that are usually non-lecythiform, sometimes sub-lecythiform. Within the traditional classification framework of Hausknecht and Krisai-Greilhuber (2007), this section is also divided into three series: ser. *Vestita*, in which the basidiospores lack a germ pore; ser. *Resinosocystidiata* Hauskn. & Krisai, in which the germ pore is indistinct but the cystidia are clavate and densely encrusted with thick resinous plaques; and ser. *Appendiculata* Hauskn. & Krisai, in which the basidiospores possess a germ pore. The subdivision of this section closely parallels that of sect. *Pholiotina*, relying primarily on the presence or absence of a germ pore and on whether the cystidia are heavily resin-encrusted. Currently, nine species (including varieties) are known worldwide in this section, of which only two have been reliably recorded from China, both from the northeastern region (Hausknecht 2009; Song 2025). Because sect. *Vestitae* is species-poor and represents a transitional group, its phylogenetic position remains unresolved, and the section appears paraphyletic (Song and Bau 2024).

Section *Intermediae* is mainly characterized by the presence of veil remnants on the pileus (only one species, *Pholiotina intermedia* (Kühner) Singer, develops an annulus) and by lecythiform cheilocystidia (Hausknecht 2009). Under the traditional classification framework of Hausknecht and Krisai-Greilhuber (2007), the section is divided into two series: ser. *Intermedia*, with lecythiform cheilocystidia and a stipe bearing an annulus; and ser. *Brunnea* Hauskn. & Krisai, also with lecythiform cheilocystidia but with veil remnants on the pileus instead of an annulus. Worldwide, 15 species are currently recognized in this section. Of these, 12 have been reliably recorded from China; notably, 11 of them were collected and described there and remain endemic to the country (Hausknecht 2009; Song and Bau 2024).

*Pholiotina* as a whole is monophyletic and aligns well with morphological classification; however, some problems persist in its infrageneric division (Tóth et al. 2013; Song and Bau 2024). For example, sect. *Vestitae* appears paraphyletic, and some species that morphologically belong to sect. *Pholiotina* fall outside its monophyletic clade; these species also receive low support, leaving their phylogenetic positions uncertain (Song and Bau 2024). Moreover, 20 known species worldwide still lack molecular sequences (Tóth et al. 2013; Song 2025). These problems can be addressed only by expanding the known species diversity of *Pholiotina*. To this end, specimens of this genus were collected between 2023 and 2025. Taxonomic examination revealed that they represent new species, which are described here to enrich the species diversity of *Pholiotina*.

## Materials and methods

### Samplings and morphological analyses

Specimens were collected from Jilin Province and the Xizang Autonomous Region, China, between 2023 and 2025. Collection and morphological examination followed the standardized protocols of Song (2025) and Tao et al. (2026). Voucher specimens are deposited in the Fungarium of Jilin Agricultural University (HMJAU/FJAU). For microscopic examination, tissue sections were rehydrated in distilled water, 5% KOH, or occasionally 1% Congo red. Lamellar reactions were tested with 25% ammonia solution (Hausknecht 2009). Observations were made and photomicrographs were taken using a Nikon Eclipse 80i microscope. Macroscopic colors were matched to the RAL color system (Reichs-Ausschuss für Lieferbedingungen und Gütesicherung; https://www.ral-guetezeichen.de/). Following Song and Bau (2025) and Song et al. (2026), microscopic measurements (e.g., of basidiospores, basidia, and cystidia) are reported as (*a*)*b*–*c*(*d*); the notation (*n*/*m*/*p*) indicates the number of spores measured from a given number of basidiomata and collections.

### DNA extraction, PCR amplification, and sequencing

DNA extraction, PCR amplification, and sequencing followed the protocols of Song and Bau (2025) and Song et al. (2026). Briefly, genomic DNA was extracted, and the ITS, 28S, and *tef*1-α regions were amplified using primer pairs ITS1F/ITS4 (White et al. 1990; Gardes and Bruns 1993), LR0R/LR7 (Vilgalys and Hester 1990; Moncalvo et al. 2000), and EF1-983F/EF1-2218R (Rehner and Buckley 2005), respectively. Reaction mixtures and cycling conditions followed the same protocols. Amplification was performed on a Bio-Rad T100™ Thermal Cycler.

PCR products were run on 1% agarose gels, purified, and sequenced by Sangon Biotech (Shanghai) Co., Ltd. The ITS region was sequenced unidirectionally, whereas 28S and *tef*1-α were sequenced bidirectionally. Raw chromatograms were edited using BioEdit v7.2.5 (Hall 1999) with the following trimming strategy: for ITS, the region from the 3' end of 18S to the 5' end of 28S was retained; for 28S and *tef*1-α, low-quality terminal regions were removed. Polymorphic sites were coded with IUPAC ambiguity codes. Sequence identities were checked via BLAST searches against the NCBI nucleotide database. All newly generated sequences were deposited in GenBank (Table 1).

**Table 1.** Details of DNA sequences used for phylogenetic reconstruction. Newly generated sequences are shown in bold; missing data are indicated by “–.” ‘T’ denotes the holotype.

**Taxon**	**Voucher specimen**	**GenBank accession numbers**			**Origin**	**References**
		**ITS**	**nrLSU**	***tef*1-α**		
*Bolbitius coprophilus*	HMJAU64958	OQ780315	OQ758216	–	China	Song and Bau 2023
*B. reticulatus*	WU30001	JX968249	JX968366	JX968455	Hungary	Tóth et al. 2013
*B. subvolvatus*	WU28379	JX968248	JX968365	JX968454	Italy	Tóth et al. 2013
*Candolleomyces leucotephrus*	SZMC-NL-1953	FM163226	FM160683	FM897219	Hungary	Nagy et al. 2011
*Conocybe alkovii*	FJAU71714	PX589399	PX589415	PX610261	China	Song et al. 2026
*C. antipus*	WU19791	JX968215	JX968332	JX968432	Austria	Tóth et al. 2013
*C. ceracea*	HMJAU64951 T	OQ758110	OQ758218	OQ758305	China	Song and Bau 2023
*C. coniferarum*	FJAU72632	PX589398	PX589414	PX610260	China	Song et al. 2026
*C. crispella*	WU27367	JX968208	JX968325	JX968426	Australia	Tóth et al. 2013
*C. cylindrospora*	HMJAU42440 T	MG250375	OQ758203	PX610262	China	Liu and Bau 2018; Song and Bau 2023; Song et al. 2026
*C. deliquescens*	HMJAU61998	OP373403	OQ758204	OQ758292	China	Song and Bau 2023
*C. enderlei*	WU21272	JX968163	JX968279	–	Italy	Tóth et al. 2013
*C. gigasperma*	SZMC-NL-3972	JX968179	JX968295	JX968403	Slovakia	Tóth et al. 2013
*C. hausknechtii*	LE253789 T	JQ247194	–	–	Russia	Malysheva 2012
*C. hornana*	SZMC-NL-3499	JX968178	JX968294	JX968402	Slovakia	Tóth et al. 2013
*C. incerta*	LE313017 T	KY614062	–	–	Russia	Malysheva 2017
*C. leporina*	SZMC-NL-2380	JX968177	JX968293	JX968401	Hungary	Tóth et al. 2013
*C. olivaceopileata*	LE313106 T	KY614059	–	–	Russia	Malysheva 2017
*C. parapilosella*	JLS3063 T	MN872706	–	–	Spain	Siquier and Salom 2020
*C. pilosella*	HMJAU64957	OQ780306	OQ758206	OQ758295	China	Song and Bau 2023
*C. praticola*	HMJAU64965	OQ780303	PX589412	PX610258	China	Song and Bau 2023; Song et al. 2026
*C. pseudocrispa*	HMJAU64944	OQ780308	OQ758211	OQ758298	China	Song and Bau 2023
*C. pubescens*	WU20759	JX968170	JX968286	JX968396	Italy	Tóth et al. 2013
*C. rostellata*	SZMC-NL-2499	JX968162	JX968278	JX968390	Sweden	Tóth et al. 2013
*C. rufostipes*	HMJAU64937 T	OQ758120	OQ758227	OQ758317	China	Song and Bau 2023
*C. semiglobata*	FJAU72755	PX589397	PX589413	PX610259	China	Song et al. 2026
*C. siennophylla*	HMJAU64966	OQ780312	OQ758210	OQ758297	China	Song and Bau 2023
*C. singeriana*	WU22129	JX968166	JX968282	JX968393	Austria	Tóth et al. 2013
*C. volvicystidiata*	LIP0001212 T	KY346827	–	–	France	Hausknecht and Broussal 2016
*C. watlingii*	WU22744	JX968172	JX968288	JX968398	Finland	Tóth et al. 2013
*Conocybula coprophila*	HMJAU62008	OR995662	OR995712	PP000855	China	Song and Bau 2024
*Co. cyanopus*	WU2134	JX968157	JX968274	JX968388	Austria	Tóth et al. 2013
*Co. longistipitata*	HMJAU64974	OR995664	OR995714	PP000857	China	Song and Bau 2024
*Co. smithii*	HMJAU62001	OP373407	OQ758215	OQ758300	China	Song and Bau 2023; Song and Bau 2024
*Con. dasypus*	HMJAU62002	OR995661	OR995711	PP000854	China	Song and Bau 2024
*Con. fuscoviolacea*	FJAU72931 T	PX612216	PX612213	PX610263	China	Song et al. 2026
*Con. lignicola*	FJAU72922 T	PX612217	PX612214	PX610264	China	Song et al. 2026
*Con. micheliana*	HMJAU65015 T	OR995677	OR994080	PP000869	China	Song and Bau 2024
*Con. ochroleuca*	HMJAU65017 T	OR995679	OR994082	PP000871	China	Song and Bau 2024
*Con. pygmaeoaffinis*	WU16600	JX968149	JX968382	–	Austria	Tóth et al. 2013
*Con. sibirica*	LE313563 T	MW682333	MW682334	–	Russia	Crous et al. 2021; Song et al. 2026
*Con. striipes*	WU26997	JX968150	JX968267	JX968383	Austria	Tóth et al. 2013; Song et al. 2026
*Con. sulcata*	SZMC-NL-1975	JX968153	JX968270	JX968386	Hungary	Tóth et al. 2013; Song et al. 2026
*Con. viscosa*	PBM3032	HQ840656	HQ840657	–	USA	Direct Submission Song et al. 2026
*Descolea antarctica*	NZ5182	AF325647	–	–	USA	Peintner et al. 2001
*D. quercina*	HMJAU64959	OQ780313	OQ758213	OQ758299	China	Song and Bau 2023
*Galerella nigeriensis*	CNF1/5859 T	JX968251	JX968368	JX968457	Nigeria	Tóth et al. 2013
*G. plicatella*	GC-07468	OQ845969	OR039001	–	Italy	Direct Submission
*Pholiotina aberrans*	SZMC-NL-3161	JX968256	JX968373	JX968459	Sweden	Tóth et al. 2013
*P. aporos*	SZMC-NL-1241	JX968260	JX968376	JX968462	Hungary	Tóth et al. 2013
*P. arrhenii*	HMJAU65103	OR995665	OR995715	PP000858	China	Song and Bau 2024
*P. bambusicola*	HMJAU65054 T	OR995681	OR994084	PP000873	China	Song and Bau 2024
*P. bifurcaticystidia*	HMJAU65030 T	OR995683	OR994086	PP000875	China	Song and Bau 2024
*P. bispora*	HMJAU65072 T	OR995686	OR994089	PP000878	China	Song and Bau 2024
*P. brevipila*	HMJAU65082 T	OR995687	OR994090	PP000879	China	Song and Bau 2024
*P. brunnea*	SZMC-NL-1216	JX968259	JX968375	JX968461	Hungary	Tóth et al. 2013
*P. calongei*	MA:Fungi 98486 T	OK256956	OK256958	–	Spain	Siquier et al. 2021
*P. changbaishanensis*	HMJAU65101 T	OR995689	OR994092	PP000881	China	Song and Bau 2024
*P. communis*	HMJAU65039 T	OR995692	OR994095	PP000884	China	Song and Bau 2024
***P. constrictibasidiata***	**FJAU78860 T**	**PZ441656**	**PZ441663**	**PZ456933**	**China**	**This study**
***P. constrictibasidiata***	**FJAU78861**	**PZ441657**	**PZ441664**	**PZ456934**	**China**	**This study**
*P. dentatomarginata*	SZMC-NL-2921	JX968258	JX968374	JX968460	Hungary	Tóth et al. 2013
*P. eburnea*	HMJAU65035 T	OR995694	OR994097	PP000886	China	Song and Bau 2024
*P. exannulata*	HMJAU45107	OR995666	OR995716	PP000859	China	Song and Bau 2024
*P. excrescenticystidiata*	HMJAU65021 T	OR995695	OR994098	PP000887	China	Song and Bau 2024
*P. filaris*	MSC378482	AY194542	–	–	USA	Hallen et al. 2003
*P. gracilenta*	PDD:87363	KM975419	KM975382	–	New Zealand	Direct Submission
*P. hadrocystis*	WU10748	MF142251	–	–	Spain	Siquier and Salom 2017
*P. horchinensis*	HMJAU65097 T	OR995697	OR994100	PP000889	China	Song and Bau 2024
*P. indica*	WU20891	JX968263	JX968378	JX968464	India	Tóth et al. 2013
*P. intermedia*	HMJAU62014	OR995667	OR995717	PP000860	China	Song and Bau 2024
***P. jilinensis***	**FJAU78873 T**	**PZ441658**	**PZ441665**	**PZ456938**	**China**	**This study**
***P. jilinensis***	**FJAU78875**	**PZ441659**	**PZ441666**	**PZ456939**	**China**	**This study**
***P. jilinensis***	**HMJAU65110**	**OR995676**	**PP001416**	**PP000868**	**China**	**Song and Bau 2024; This study**
*P. liudingshanensis*	HMJAU65053 T	OR995700	OR994103	PP000892	China	Song and Bau 2024
*P. longicystidiata*	HMJAU65060 T	OR995702	OR994105	PP000894	China	Song and Bau 2024
*P. mediterranea*	MA:Fungi 89945 T	MF142244	–	–	Spain	Siquier and Salom 2017
*P. micropora*	HMJAU65080 T	OR995704	OR994107	PP000896	China	Song and Bau 2024
*P. parvula*	GLM-F39727	MK412362	–	–	Germany	Direct Submission
*P. pauli*	LUG 19803 T	OQ544423	OQ544425	–	France	Musumeci 2023
*P. pseudoampullaceocystis*	AK20191010 T	MT903471	–	–	Germany	Karich 2020
***P. raribasidiata***	**FJAU78862 T**	**PZ441661**	**PZ441667**	**PZ456936**	**China**	**This study**
***P. raribasidiata***	**FJAU78868**	**PZ441662**	**PZ441668**	**PZ456937**	**China**	**This study**
***P. raribasidiata***	**FJAU78870**	**PZ441660**	**PZ441669**	**PZ456935**	**China**	**This study**
*P. rostellulata*	HMJAU65050 T	OR995706	OR994109	PP000898	China	Song and Bau 2024
*P. rufidispora*	HMJAU65027 T	OR995707	OR994110	PP000899	China	Song and Bau 2024
*P. serrata*	HMJAU62006	OP538570	OQ758217	OQ758301	China	Song and Bau 2023
*P. sphagnicola*	HMJAU64971 T	PP034263	PP034265	PP034744	Mongolia	Burenbaatar et al. 2024
*P. sulciceps*	HMJAU65100 T	OR995710	OR994113	PP000902	China	Song and Bau 2024
*P. teneroides*	SZMC-NL-3501	JX968264	JX968379	JX968465	Slovakia	Tóth et al. 2013
*P. utricystidiata*	HMJAU45885	OR995668	OR995718	PP000861	China	Song and Bau 2024
*P. vestita*	SZMC-NL-2191	JX968266	JX968381	JX968467	Hungary	Tóth et al. 2013
*P. vexans*	HMJAU45078	OR995669	OR995719	PP000862	China	Song and Bau 2024
*Psathyrella piluliformis*	HMJAU37922	MG734716	MW413364	MW411001	China	Yan and Bau 2018

### Sequence alignment and phylogenetic analyses

Phylogenetic analyses followed the protocols of Song and Bau (2025) and Song et al. (2026). Sequences were retrieved from GenBank (Table 1) and supplemented with 21 newly generated sequences. Multiple sequence alignments of ITS, 28S, and *tef*1-α were generated using the MAFFT online service (Katoh et al. 2019) and manually refined in MEGA7 (Kumar et al. 2016) by trimming ambiguous 5' and 3' ends. The alignments were then concatenated into a combined dataset with PhyloSuite v2 (Zhao et al. 2025). Optimal partitioning schemes and nucleotide substitution models were selected using ModelFinder v2.2.0 (Kalyaanamoorthy et al. 2017). Maximum likelihood (ML) analysis was conducted in IQ-TREE 3 with 1,000 standard bootstrap replicates (Nguyen et al. 2015; Wong et al. 2025). Bayesian inference (BI) was run in MrBayes v3.2.7a for 1,000,000 generations, and convergence was confirmed when the average standard deviation of split frequencies fell below 0.01 (final value 0.008) (Ronquist et al. 2012). Phylogenetic trees were visualized and edited using iTOL (Letunic and Bork 2019), Adobe Photoshop 2021, and Adobe Illustrator 2021. Species of *Psathyrella* (Fr.) Quél. and *Candolleomyces* D. Wächt. & A. Melzer (Song and Bau 2023) were used as outgroups.

### Abbreviations

For Latin names: *B.* = *Bolbitius*; *C.* = *Conocybe*; *Ca.* = *Candolleomyces*; *Co.* = *Conocybula*; *Con.* = *Conobolbitina*; *D.* = *Descolea*; *G.* = *Galerella*; *P.* = *Pholiotina*; *Ps.* = *Psathyrella*.

## Results

### Phylogenetic analyses

**Figure 1.**
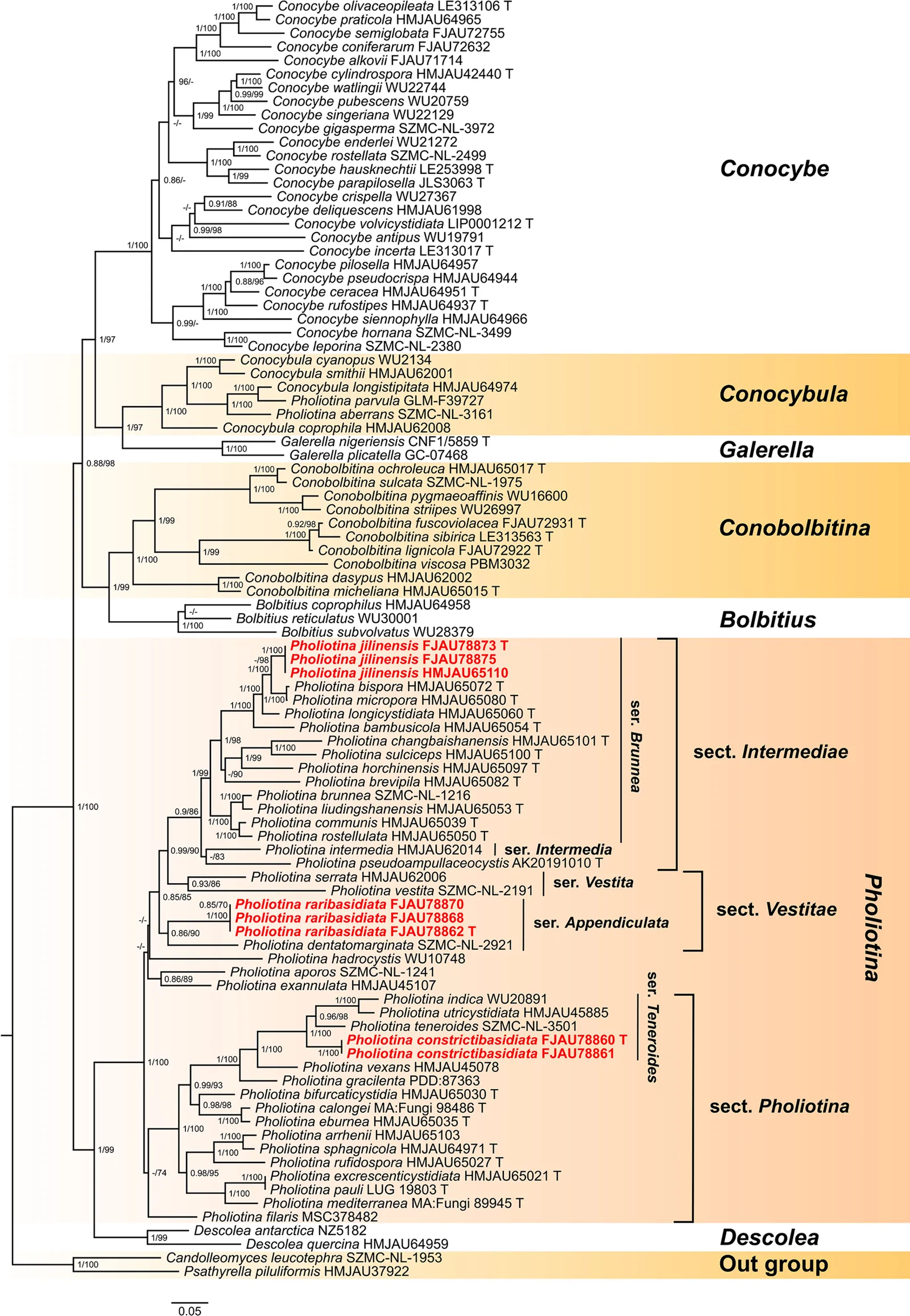
Phylogenetic placement of *Pholiotina* within the *Bolbitiaceae* inferred through Bayesian inference and maximum likelihood analyses of a combined ITS, 28S, and *tef*1-α dataset. Newly described species proposed in this study are indicated in red bold. T = holotype.

Phylogenetic trees were inferred using Bayesian inference and maximum likelihood (ML), based on a concatenated dataset of ITS, 28S, and *tef*1-α sequences. The ML tree is shown in Fig. 1. Its topology is nearly identical to that of the Bayesian tree, differing only at poorly supported branches. Both trees were deposited in the Zenodo repository (see Data availability). Support values (Bayesian posterior probabilities ≥ 0.85 and ML bootstrap ≥ 70%) are shown at the nodes; values below these thresholds are indicated by a dash (Fig. 1).

The aligned lengths of ITS, 28S, and *tef*1-α were 893 bp, 1,308 bp, and 1,107 bp, respectively. The concatenated matrix contained 94 sequences and 3,308 columns, with 1,569 distinct patterns, 1,149 parsimony-informative sites, 206 singleton sites, and 1,952 constant sites. For both maximum likelihood and Bayesian analyses, best-fit partition models (edge-unlinked) were selected in ModelFinder, using the AIC criterion for ML and BIC for Bayesian inference. Based on AIC, the selected models were GTR+F+R5 for ITS, GTR+F+I+R3 for 28S, and GTR+R6 for *tef*1-α. Under BIC, the models were GTR+F+I+G4 for ITS and 28S and SYM+I+G4 for *tef*1-α.

The phylogenetic tree (Fig. 1) recovered *Pholiotina* as a strongly supported monophyletic clade (PP/MLbs = 1/99). Both sect. *Pholiotina* and sect. *Intermediae* formed strongly supported monophyletic groups (1/100 and 0.99/90, respectively). Within sect. *Pholiotina*, only ser. *Teneroides* was resolved as a strongly supported monophyletic lineage (1/100), while the remaining series were paraphyletic or polyphyletic. In sect. *Intermediae*, both ser. *Intermedia* and ser. *Brunnea* were monophyletic, with support values of –/83 and 0.9/86, respectively. By contrast, sect. *Vestitae* appeared paraphyletic, comprising two separate monophyletic clades: ser. *Vestita* (0.99/90) and ser. *Appendiculata* (0.85/85). Sequence data are available for only five species in this section, so more extensive sampling is needed to clarify its circumscription. Within *Pholiotina*, the phylogenetic placements of *P. hadrocystis* (Kits van Wav.) Courtec. (WU10748), *P. aporos* (Kits van Wav.) Clémençon (SZMC-NL-1241), *P. exannulata* (Kühner & Watling) M.M. Moser ex Courtec. (HMJAU45107), and *P. pseudoampullaceocystis* Karich remain unresolved due to low node support, likely reflecting limited taxon sampling. Their positions require further investigation.

In sect. *Pholiotina* ser. *Teneroides*, specimens FJAU78860 and FJAU78861 formed a distinct monophyletic clade with full support (PP/MLbs = 1/100). This clade was sister to the monophyletic group containing *P. teneroides* (J.E. Lange) Singer (SZMC-NL-3501), *P. utricystidiata* Enderle & H.-J. Hübner (HMJAU45885), and *P. indica* K.A. Thomas, Hauskn. & Manim. (WU20891), also with full support (1/100). In sect. *Vestitae* ser. *Appendiculata*, specimens FJAU78862, FJAU78868, and FJAU78870 formed a strongly supported monophyletic clade (1/100), which grouped as sister to the clade containing *P. dentatomarginata* (Watling) Enderle (SZMC-NL-2921) with 0.86/90 support. In sect. *Intermediae* ser. *Brunnea*, specimens FJAU78873, FJAU78875, and HMJAU65110 formed a fully supported monophyletic clade (1/100), sister, again with full support (1/100), to the clade comprising *P. bispora* T. Bau & H.B. Song (HMJAU65072) and *P. micropora* T. Bau & H.B. Song (HMJAU65080). Additionally, BLAST searches of their ITS sequences against the NCBI database returned the following similarity values, sorted in descending order: specimen FJAU78860 showed 90.94% similarity to *P. indica* (WU20891), 90.58% to *P. utricystidiata* (HMJAU45885), and 89.92% to *P. teneroides* (SZMC-NL-3501); specimen FJAU78862 showed 86.48% similarity to *P. dentatomarginata* (SZMC-NL-2921), 85.5% to *P. serrata* (T. Bau & Jing Liu) T. Bau & H.B. Song (HMJAU42442), and 85.41% to *P. exannulata* (HMJAU45107); specimen FJAU78873 showed 92.61% similarity to *P. longicystidiata* T. Bau & H.B. Song (HMJAU65060), 92.49% to *P. micropora* (HMJAU65080), and 91.89% to *P. bispora* (HMJAU65072).

Thus, based on morphological and phylogenetic evidence and their distinct phylogenetic positions, three new species are proposed: *P. constrictibasidiata* (represented by specimens FJAU78860 and FJAU78861), *P. raribasidiata* (represented by specimens FJAU78862, FJAU78868, and FJAU78870), and *P. jilinensis* (represented by specimens FJAU78873, FJAU78875, and HMJAU65110).

### Taxonomy

#### Pholiotina constrictibasidiata

Taxon classificationFungiAgaricalesBolbitiaceae

H.B. Song & J.L. Deng sp. nov.

6AAF1064-2B2E-534A-98B4-BC33178F2057

863841

**Figure 2.**
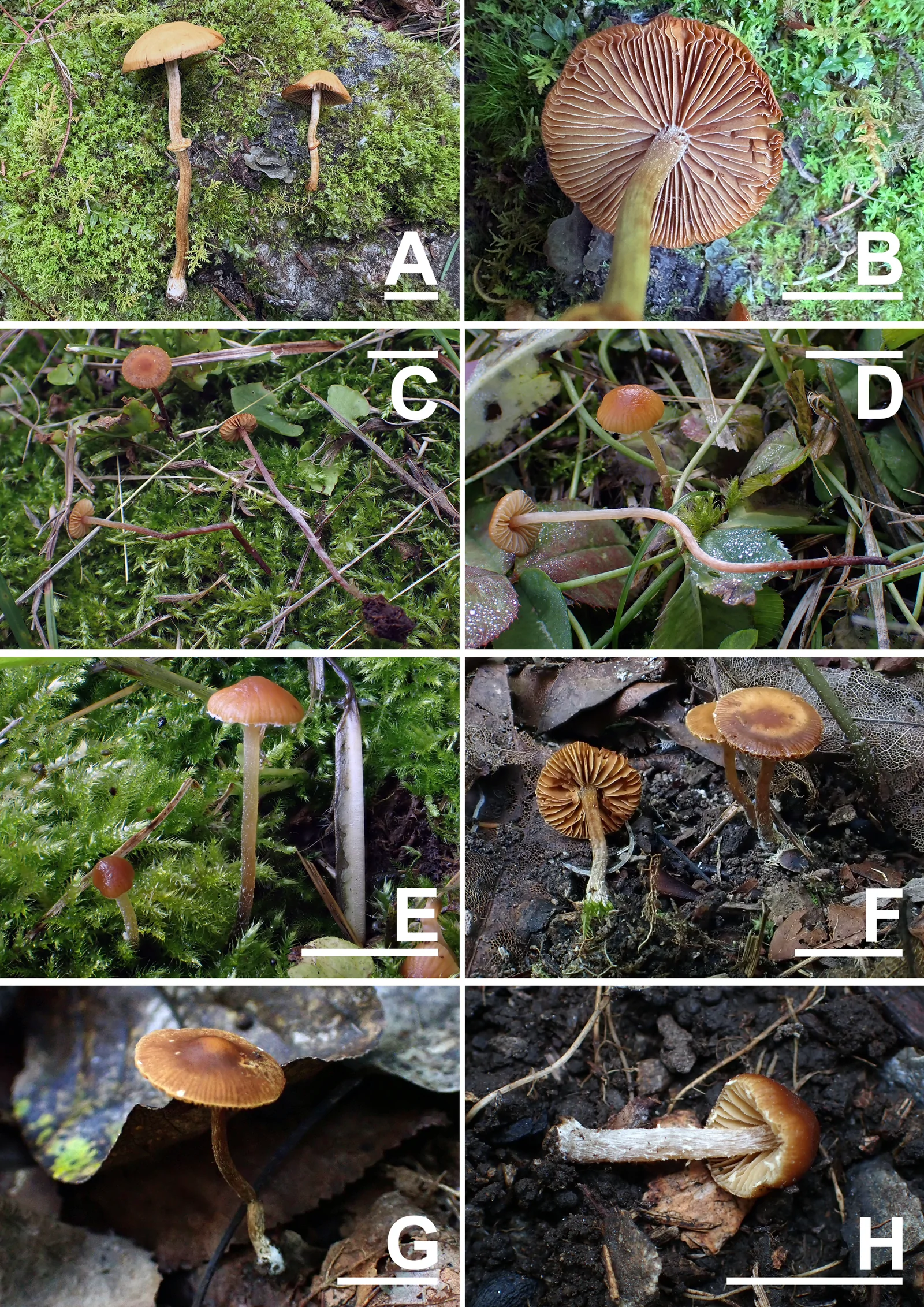
Basidiomata of *Pholiotina* species. **A, B**. *P. constrictibasidiata* (FJAU78860 T); **C**. *P. raribasidiata* (FJAU78862 T); **D**. *P. raribasidiata* (FJAU78868); **E**. *P. raribasidiata* (FJAU78867); **F**. *P. jilinensis* (FJAU78873 T); **G**. *P. jilinensis* (FJAU78874); **H**. *P. jilinensis* (FJAU78875). Scale bars: 1 cm; T = holotype.

**Figure 3.**
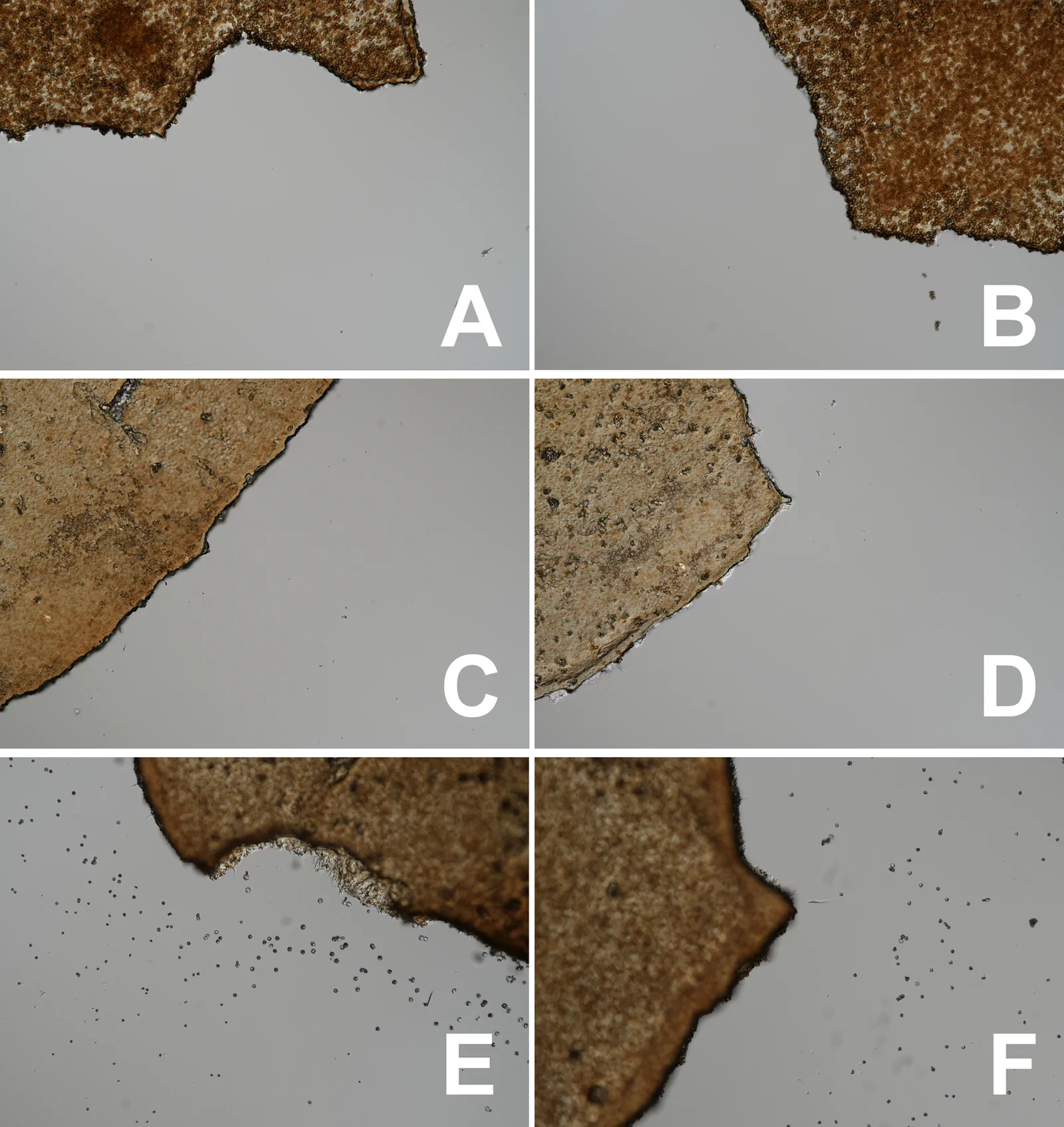
Illustration of the ammonia reaction in *Pholiotina* species. **A, B**. *P. constrictibasidiata* (FJAU78860, T); **C, D**. *P. raribasidiata* (FJAU78862, T); **E, F**. *P. jilinensis* (FJAU78873, T). T = holotype.

**Figure 4.**
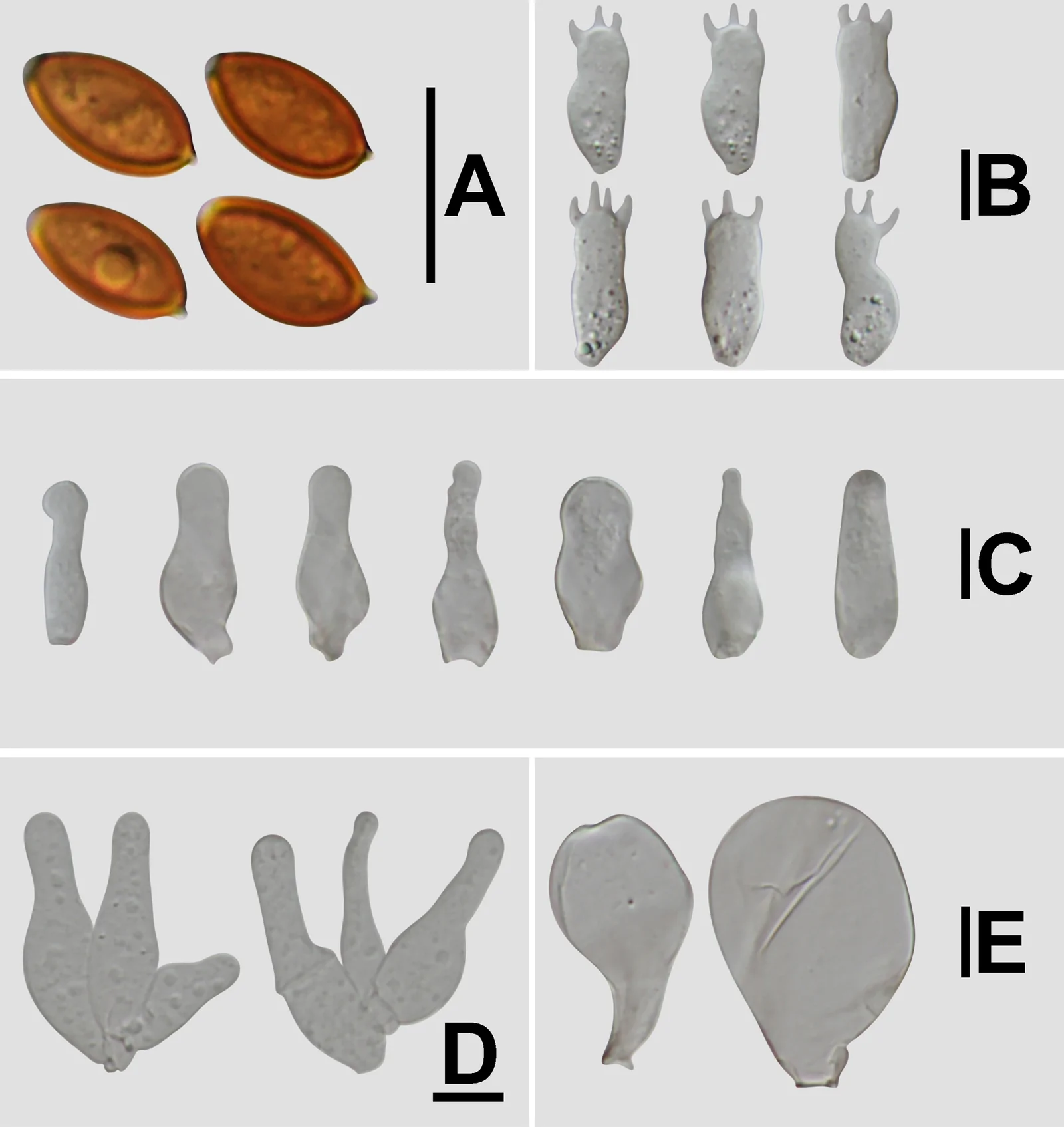
Microscopic structures of *Pholiotina constrictibasidiata* (FJAU78860). **A**. Basidiospores; **B**. Basidia; **C**. Cheilocystidia; **D**. Caulocystidia; **E**. Pileipellis elements. Scale bars: 10 μm (**A–E**).

**Figure 5.**
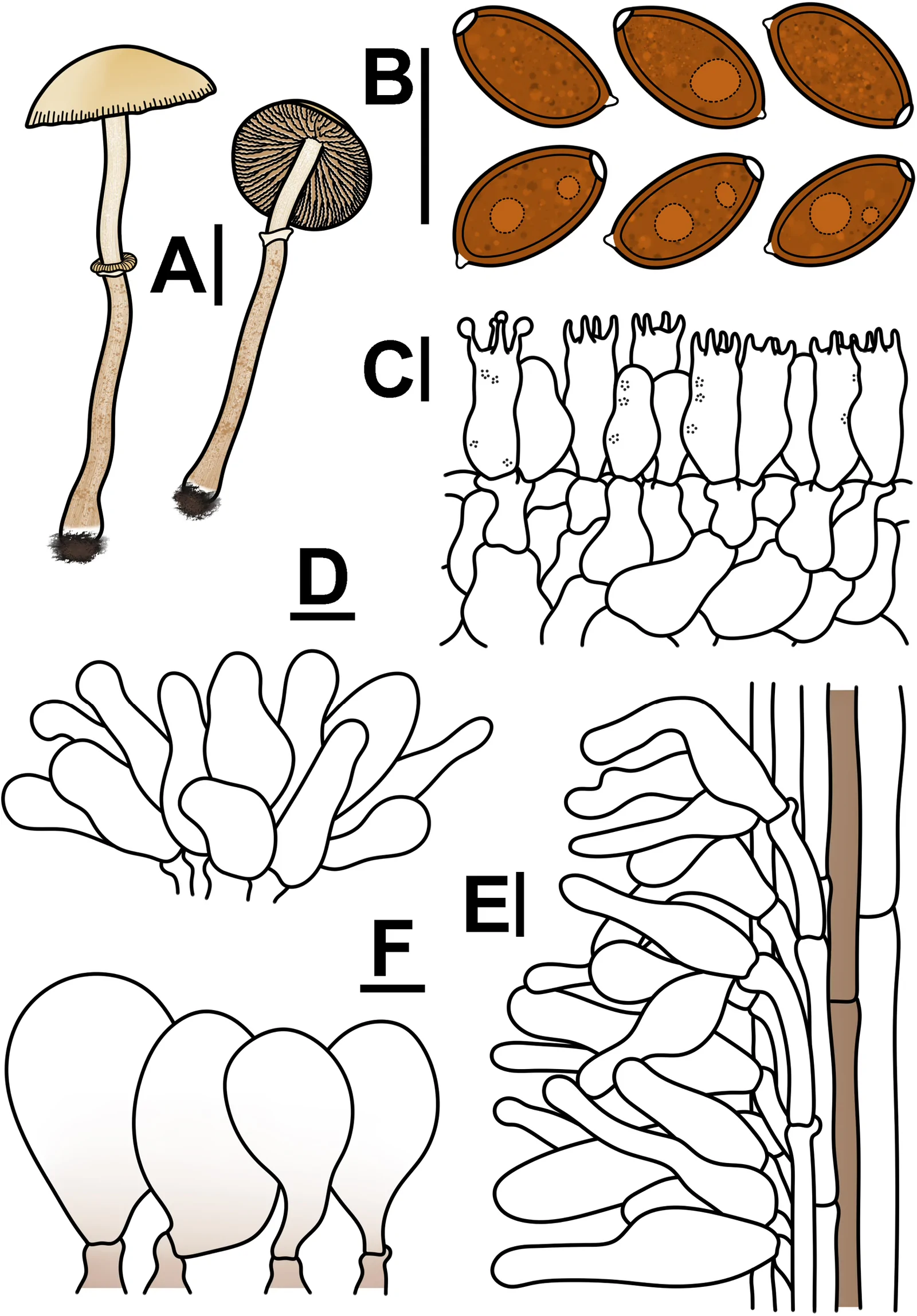
*Pholiotina constrictibasidiata* (FJAU78860). **A**. Basidiomata; **B**. Basidiospores in 5% KOH solution; **C**. Hymenium and subhymenium; **D**. Cheilocystidia; **E**. Stipitipellis; **F**. Pileipellis elements. Scale bar: 1 cm (**A**); 10 μm (**B–F**).

Figs 2A, 2B, 3A, 3B, 4, 5

##### Etymology.

The specific epithet “constrictibasidiata” refers to basidia that are subutriform to cylindrical, with a median constriction.

##### Holotypus.

China • Xizang Autonomous Region, Xigazê City, Chomo County, 24 July 2025, 27°37'12"N, 88°56'24"E, alt. 3,610 m, Song-ming Tang, 2025072417 (FJAU78860).

##### Diagnosis.

The cheilocystidia of this species are predominantly utriform, making it easily confused with species of ser. *Teneroides*, however, most of its basidia are subutriform to cylindrical with a median constriction, which distinguishes it from other species in ser. *Teneroides*.

##### Description.

***Basidiomata*** mycenoid. ***Pileus*** 1.5–2.5 cm diameter, first hemispherical to obtusely conical, later convex to plano-convex, margin straight, undulate to eroded. Surface beige (RAL 1001), sand yellow (RAL 1002) to ochre brown (RAL 8001). Hygrophanous, smooth, glabrous, margin short striate, without veil remnants. ***Context*** thin, light ivory (RAL 1015) to ochre brown (RAL 8001), no specific odor or taste. ***Lamellae*** adnexed to narrowly adnate, ventricose, crowded, unequal length, brown beige (RAL 1011), ochre brown (RAL 8001) to clay brown (RAL 8003), serrulate white at edge. ***Stipe*** 3–6 cm long, 2–4 mm thick, cylindrical, upper part light ivory (RAL 1015) to beige (RAL 1001), lower part clay brown (RAL 8003) to fawn brown (RAL 8007), surface pruinose, with longitudinal fibrillose striae, base subbulbous. ***Ring*** at the middle, membranous, same color as stipe, upper surface with undulate sulcate, lower surface smooth.

***Basidiospores*** (40/2/2) 9–10.4(–10.8) × (4.8–)5.1–6(–6.5) μm, *Q* = (1.61–)1.65–1.9(–1.96), *Q_m_* = 1.78(±0.08), non-lentiform, oblong, slightly amygdaliform, thick-walled, oil droplets present, germ pore diameter 1–2 μm, ochre brown (RAL 8001) to copper brown (RAL 8004) in KOH solution. ***Basidia*** (18–)20–26 × (6–)7–10 μm, utriform, subclavate to cylindrical, with median constriction, 4-spored, sterigmata 3–6 μm long, with vacuolar contents in basidia. ***Cheilocystidia*** (21–)22–38(–39) × (6–)7–15 μm, mainly utriform, narrowly utriform to broadly utriform, capitate, clavate, nearly spheropedunculate, subcylindrical, narrowly conical, lageniform, some with slightly swollen apex, lamellae edge sterile. ***Pleurocystidia*** absent. ***Caulocystidia*** (16–)20–48(–50) × 6–14 μm, mixed, cylindrical, narrowly utriform to utriform, nearly nettle hair-shaped, lageniform to long-necked lageniform, some with slightly swollen apex. ***Pileipellis*** epithelioid hymeniderm, composed of spheropedunculate cells 24–50(–51) × (14–)15–27(–30) μm, base with fawn brown (RAL 8007) pigment. ***Pileocystidia*** absent. All structures have clamp connections. Negative reaction with ammonia.

##### Habitat.

Scattered growth on plateau grasslands in summer.

##### Known distribution.

Xizang Autonomous Region, Xigazê City, China.

##### Additional collection examined.

China • Xizang Autonomous Region, Xigazê City, Chomo County, 24 July 2025, 27°37'12"N, 88°56'24"E, alt. 3,610 m, Song-ming Tang, 5A21 (FJAU78861). Sequences were obtained from all specimens mentioned above, and their morphology was also examined.

##### Notes.

Because the stipe bears a ring, this species belongs to sect. *Pholiotina*, and because its cheilocystidia are utriform, it is placed in ser. *Teneroides* (Hausknecht and Krisai-Greilhuber 2007). Within ser. *Teneroides*, it differs from *P. teneroides* in that the latter has slightly larger basidiospores, up to 14 μm, and 2-spored basidia (Singer 1936). It differs from *P. utricystidiata* by the latter’s superior ring and clavate basidia (Enderle and Hübner 1999). From *P. tucumana* Singer, it is distinguished by the latter’s campanulate pileus, ventricose basidia, and lack of a basal bulb (Singer 1973). It is separated from *P. altaica* Singer by the latter’s 2-spored basidia and cheilocystidia with crystals at the apex (Singer 1950). Compared with *P. flexipes* (Watling) Enderle, the latter has a superior ring and basidiospores with a germ pore less than 1 μm in diameter (Enderle 1997; Hausknecht et al. 2004). It differs from *P. stercoraria* (Watling) Enderle, which grows on horse dung, has a ring without striae but with flocculose material below, and basidiospores with a germ pore approximately 1 μm in diameter (Enderle 1997; Hausknecht et al. 2004). It differs from *P. indica*, which grows on the dung of *Elephas maximus* Linnaeus, has a superior ring, and clavate basidia (Thomas et al. 2001). It differs from *P. procera* Singer, which has a larger pileus, up to 5 cm in diameter, context with a faint farinaceous odor, and a small basidiospore germ pore (Singer 1950). Phylogenetically, the independent lineage of this species is clearly distinct from *P. utricystidiata*, *P. teneroides*, and *P. indica*, and the clade comprising these four species is sister to *P. vexans* (P.D. Orton) Bon. However, *P. vexans* belongs to ser. *Vexans* and has a different type of cheilocystidia, making the two taxa easily distinguishable. (Bon 1991).

#### Pholiotina raribasidiata

Taxon classificationFungiAgaricalesBolbitiaceae

H.B. Song & J.L. Deng sp. nov.

520FA4DD-79B4-5E61-A257-63F11C95A2EA

863842

**Figure 6.**
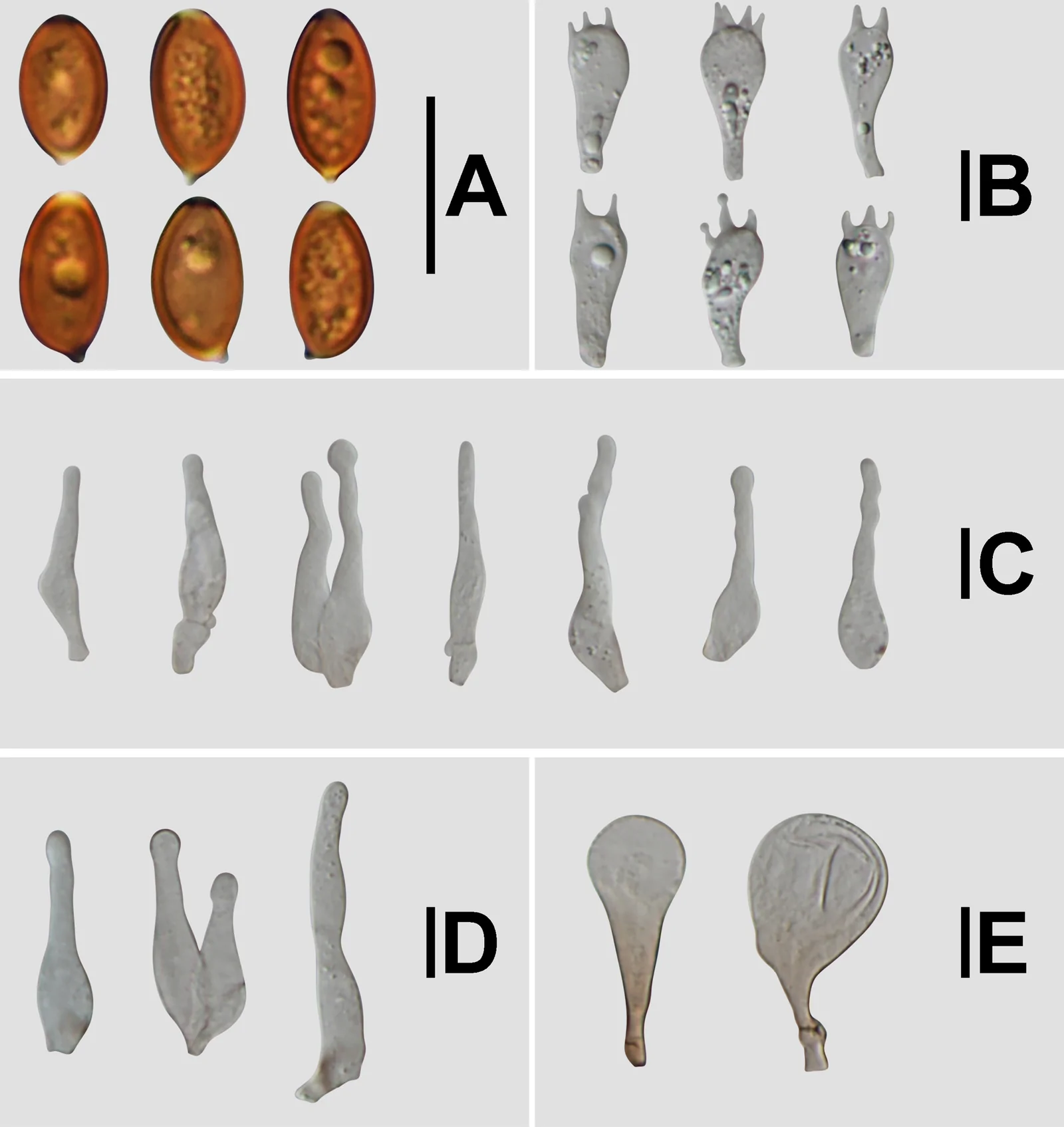
Microscopic structures of *Pholiotina raribasidiata* (FJAU78862). **A**. Basidiospores; **B**. Basidia; **C**. Cheilocystidia; **D**. Caulocystidia; **E**. Pileipellis elements. Scale bars: 10 μm (**A–E**).

**Figure 7.**
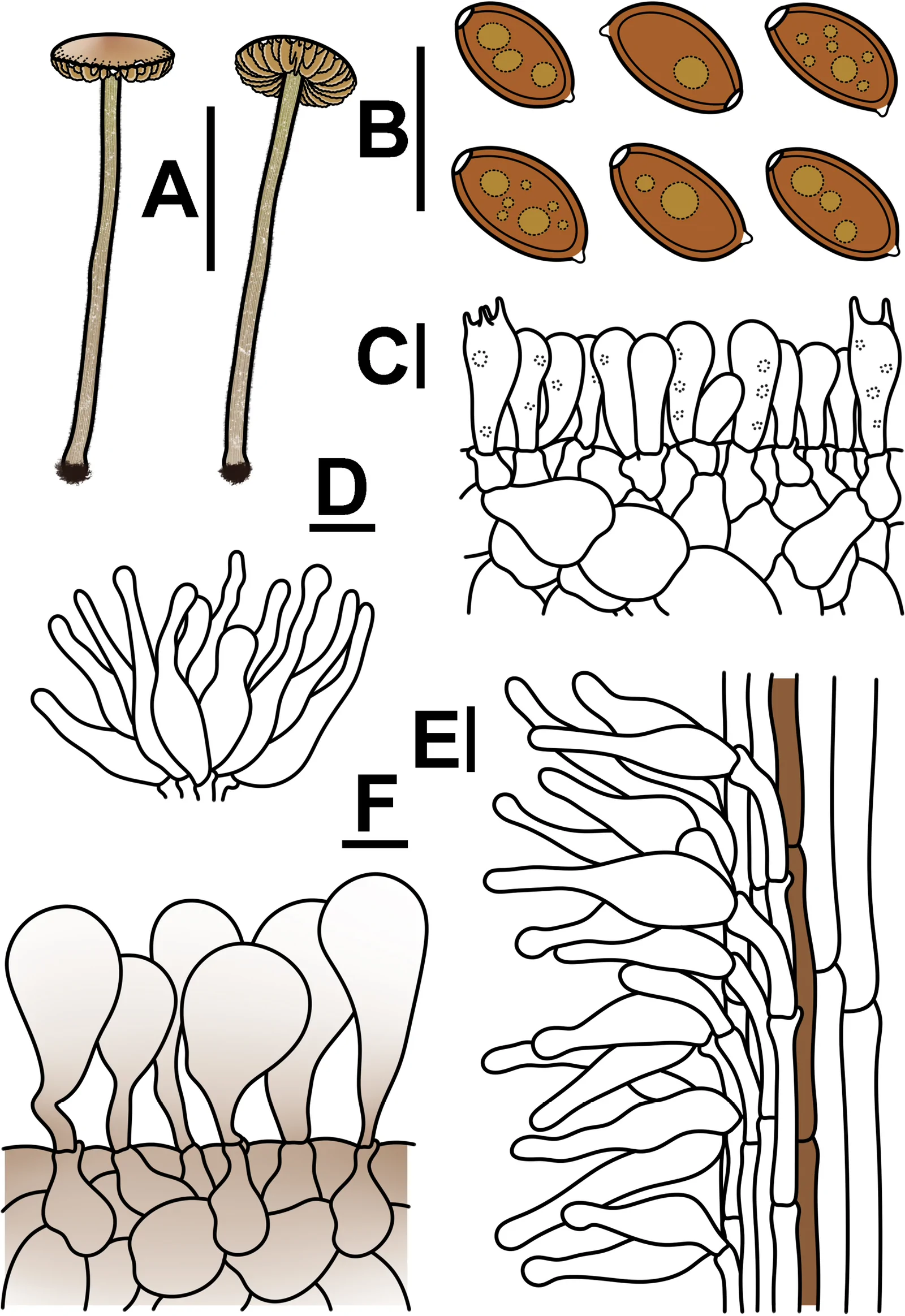
*Pholiotina raribasidiata* (FJAU78862). **A**. Basidiomata; **B**. Basidiospores in 5% KOH solution; **C**. Hymenium and subhymenium; **D**. Cheilocystidia; **E**. Stipitipellis; **F**. Pileipellis. **A**. Scale bar = 1 cm; **B–F**. Scale bars: 10 μm.

Figs 2C, 2D, 2E, 3C, 3D, 6, 7

##### Etymology.

The specific epithet “raribasidiata” refers to basidia being rare.

##### Holotypus.

China • Jilin Province, Changchun City, Xincheng Street, 20 September 2024, 43°48'43"N, 125°24'15"E, alt. 221 m, Ji-li Deng, S24092001 (FJAU78862).

##### Diagnosis.

The main distinguishing features of this species are basidiospores and basidia both rare (ratio of basidia to basidioles less than 1/20), pileus applanate when mature, cheilocystidia long-necked lageniform to nettle hair-shaped, some with swollen apex, sub-lecythiform. Based on these features, it can be distinguished from other species in sect. *Vestitae*.

##### Description.

***Basidiomata*** mycenoid. ***Pileus*** 0.5–1.5 cm in diameter, at first hemispherical to subcampanulate, later convex to applanate, margin straight, slightly undate. Center fawn brown (RAL 8007) to mahogany brown (RAL 8016), margin pastel yellow (RAL 1034) to sandy yellow (RAL 1002), ochre brown (RAL 8001). Hygrophanous, surface smooth, glabrous, when moist striate extending to pileus center, initial veil remains at the edge of the pileus, gradually disappearing. ***Context*** thin, light ivory (RAL 1015) to ivory (RAL 1014), with no specific odor or taste. ***Lamellae*** adnexed to narrowly adnate, ventricose, slightly loosely, unequal length, light ivory (RAL 1015) to ivory (RAL 1014), ochre brown (RAL 8001) to clay brown (RAL 8003), with a serrulate margin. ***Stipe*** 2–5 cm long, 0.5–2.5 mm thick, cylindrical, upper part light ivory (RAL 1015) to sand yellow (RAL 1002), pale brown (RAL 8025), lower part red brown (RAL 8012) to chestnut brown (RAL 8015), mahogany brown (RAL 8016), surface pruinose and pubescent, longitudinally fibrillose-striate, base subbulbous.

***Basidiospores*** (60/3/3) (7.2–)7.6–10.3(–11) × (4.3–)4.4–5.7(–5.9) μm, *Q* = (1.53–)1.58–1.88(–1.92), *Q_m_* = 1.75(±0.09), slightly lentiform, slightly ellipsoid to oblong, thick-walled, oil droplets present, germ pore diameter 0.5–2 μm, some eccentric, basidiospores clay brown (RAL 8003) to fawn brown (RAL 8007) in KOH solution. ***Basidia*** 18–24(–26) × 8–10 μm, rare (ratio of basidia to basidioles less than 1/20), clavate, 4(2)-spored, sterigmata 3–6 μm long, with vacuolar contents. ***Cheilocystidia*** 23–41 × 6–9(–10) μm, long-necked lageniform to nettle hair-shaped, some with swollen apex, sub-lecythiform, lamellae edge sterile. ***Pleurocystidia*** absent. ***Caulocystidia*** (22–)23–53(–55) × 7–14(–15) μm, similar to cheilocystidia, long-necked lageniform to nettle hair-shaped, some with swollen apex, sub-lecythiform. ***Pileipellis*** epithelioid hymeniderm, composed of spheropedunculate cells (20–)21–51(–55) × (11–)13–30 μm, base with nut brown (RAL 8011) pigment. ***Pileocystidia*** absent. All structures have clamp connections. Negative reaction with ammonia.

##### Habitat.

In autumn, scattered in the moss layer of grasslands.

##### Known distribution.

Jilin Province, Changchun City, China.

##### Additional material examined.

China • Jilin Province, Changchun City, Xincheng Street, 18 September 2024, 43°48'43"N, 125°24'17"E, alt. 220 m, Ji-li Deng, SH24091803 (FJAU78863), SH24091804 (FJAU78864), SH24091805 (FJAU78865), SH24091806 (FJAU78866). China • Jilin Province, Changchun City, Xincheng Street, 19 September 2024, 43°48'43"N, 125°24'16"E, alt. 221 m, Han-bing Song, S24091901 (FJAU78867), S24091902 (FJAU78868), S24091903 (FJAU78869). China • Jilin Province, Changchun City, Xincheng Street, 20 September 2024, 43°48'43"N, 125°24'15"E, alt. 220 m, Ji-li Deng, S24092003 (FJAU78870). China • Jilin Province, Changchun City, Xincheng Street, 29 September 2024, 43°48'43"N, 125°24'15"E, alt. 220 m, Han-bing Song, SC24092902 (FJAU78871), S24092903 (FJAU78872). Sequences were obtained from all specimens mentioned above, and their morphology was also examined.

##### Notes.

Because the pileus bears a veil, the cheilocystidia are non-lecythiform, and the basidiospores have a germ pore, this species belongs to sect. *Vestitae* ser. *Appendiculata* (Hausknecht and Krisai-Greilhuber 2007). Within ser. *Appendiculata*, it can be distinguished from *P. velata* (Velen.) Hauskn. by the latter’s slightly sour taste and basidiospores averaging less than 8 μm (Hausknecht 1999). It differs from *P. exannulata*, which has a pileus up to 3 cm in diameter, dense to very dense lamellae, and cheilocystidia that are mostly lageniform with a relatively short neck (Courtecuisse 1985). It is separated from *P. exannulata* var. *maculata* Hauskn. by the latter’s pileus up to 5 cm in diameter, stipe base up to 1 cm in diameter, and growth on decaying rice straw in paddy fields (Hausknecht 2007). It differs from *P. nemoralis* (Harmaja) Bon, which has moderately crowded to crowded lamellae and ovoid basidiospores up to 14 μm (Bon 1991). Phylogenetically, this species is closely related to *P. dentatomarginata*; however, the latter has a pileus up to 4.7 cm in diameter, initially almost glandiform and later conical-campanulate with a broad umbo, and crowded lamellae, characters that make the two species readily distinguishable (Hausknecht 2007).

#### Pholiotina jilinensis

Taxon classificationFungiAgaricalesBolbitiaceae

H.B. Song & J.L. Deng sp. nov.

08DA61CE-6664-59E1-B1DD-34D5553059B6

863843

**Figure 8.**
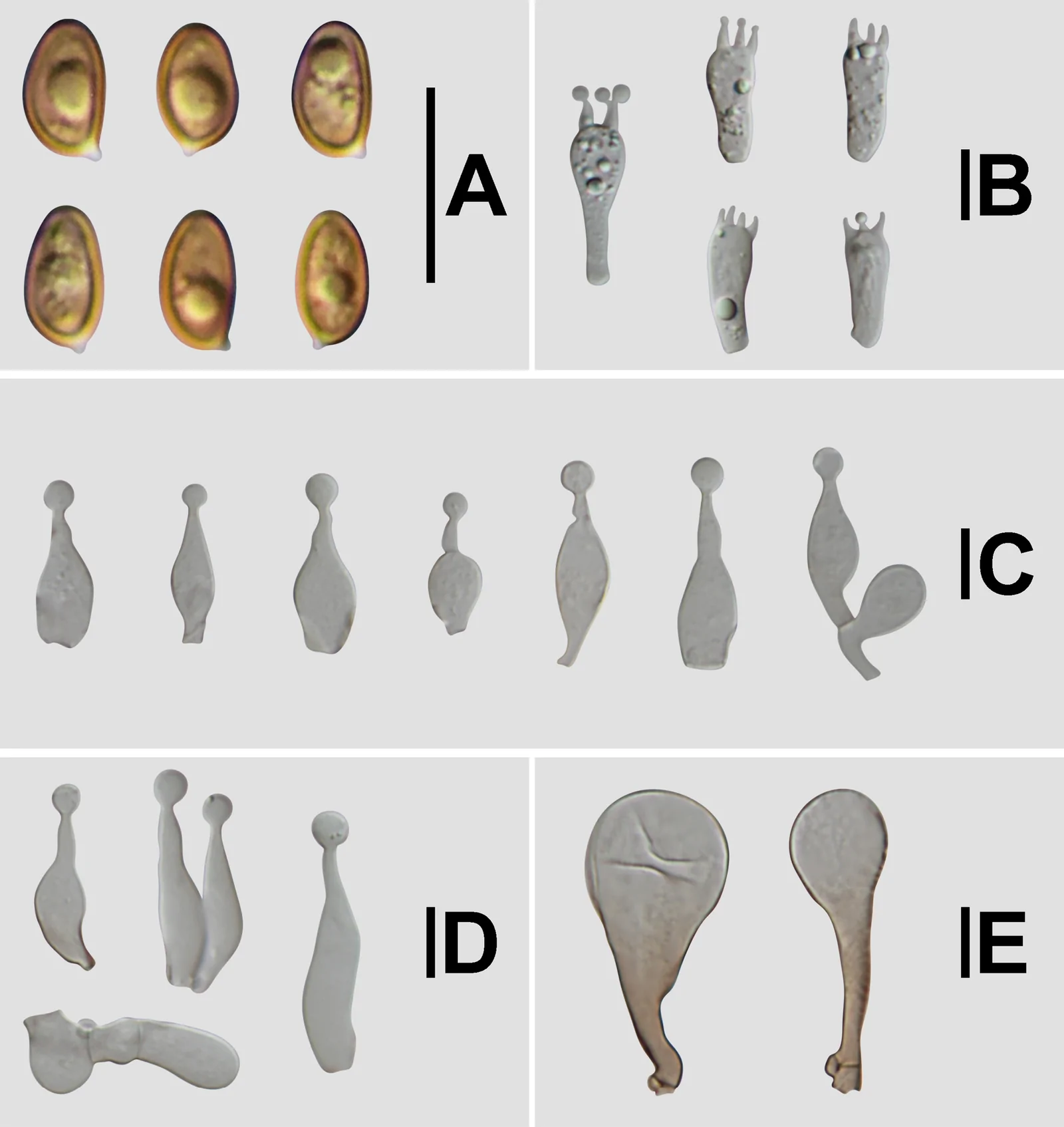
Microscopic structures of *Pholiotina jilinensis* (FJAU78873). **A**. Basidiospores; **B**. Basidia; **C**. Cheilocystidia; **D**. Caulocystidia; **E**. Pileipellis elements. Scale bars: 10 μm (**A–E**).

**Figure 9.**
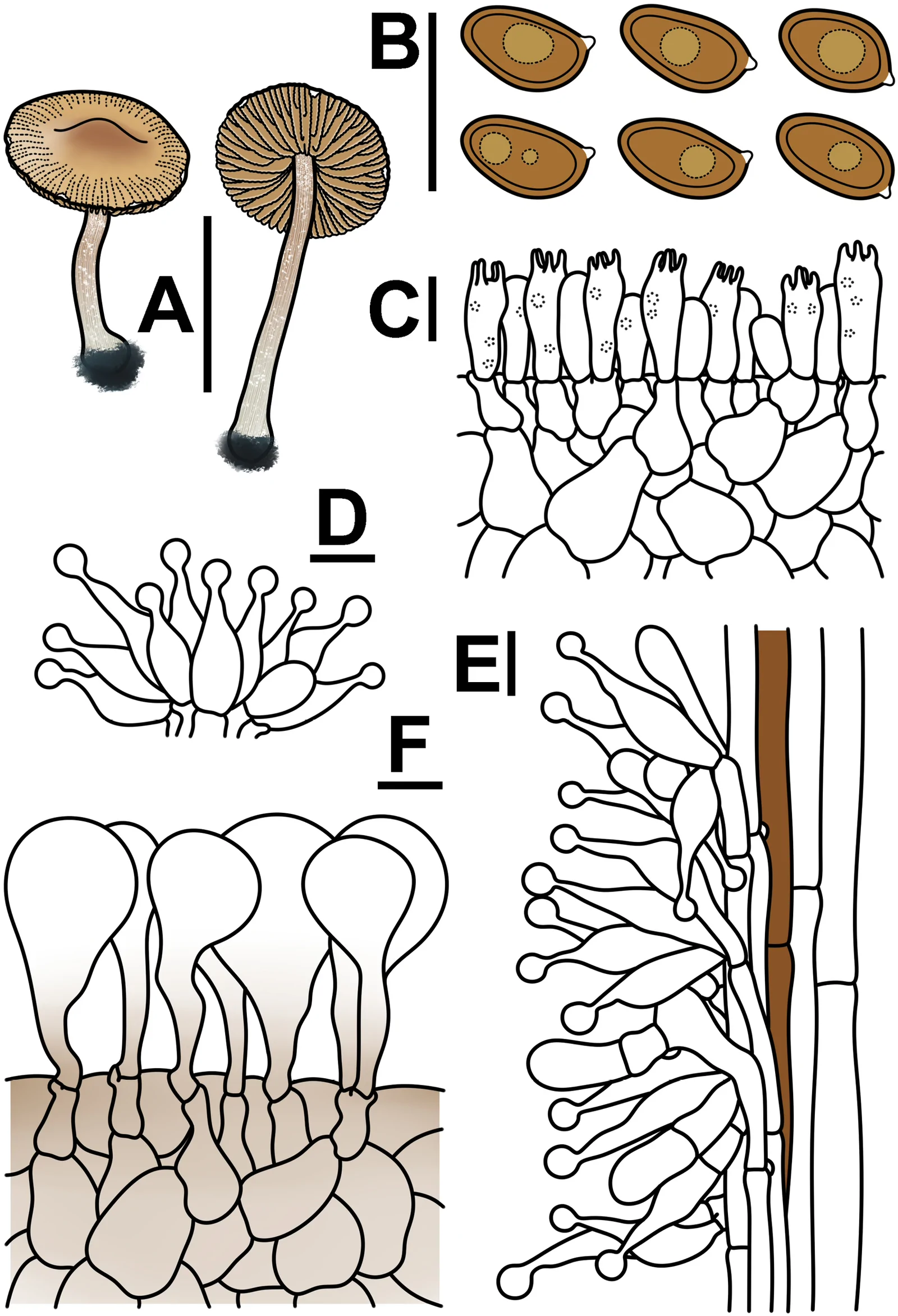
*Pholiotina jilinensis* (FJAU78873). **A**. Basidiomata; **B**. Basidiospores in 5% KOH solution; **C**. Hymenium and subhymenium; **D**. Cheilocystidia; **E**. Stipitipellis; **F**. Pileipellis. Scale bar: 1 cm (**A**); 10 μm (**B–F**).

Figs 2F, 2G, 2H, 3E, 3F, 8, 9

##### Etymology.

The epithet “jilinensis” refers to the type locality, Jilin City, where the holotype of this species was collected.

##### Holotypus.

China • Jilin Province, Jilin City, Jiaohe City, Red Leaf Valley, 18 August 2024, 43°42'17"N, 127°4'29"E, alt. 528 m, Han-bing Song, 4J10 (FJAU78873).

##### Diagnosis.

The basidiospores of this species are without germ pore, and the species was collected from Jilin Province. It is easily confused with *P. serrata*, which also comes from Jilin Province and lacks a germ pore, but *P. serrata* has non-lecythiform cheilocystidia, basidiospores 7–9 μm, and grows in moss layers in grasslands, thus can be distinguished from it.

##### Description.

***Basidiomata*** mycenoid. ***Pileus*** 0.5–1.5 cm, initially obtusely conical to nearly campanulate, then subumbonate to applanate or slightly depressed with a low broad umbo, margin straight, slightly undulate. When young pileus red brown (RAL 8012) to chestnut brown (RAL 8015), when mature olive brown (RAL 8008) to nut brown (RAL 8011), ochre brown (RAL 8001). Surface hygrophanous, smooth, glabrous, when moist slightly viscid, margin striate halfway to center, initial veil remains at the edge of the pileus, gradually disappearing. ***Context*** thin, concolorous with pileus, with no specific odor or taste. ***Lamellae*** adnexed to narrowly adnate, ventricose, moderately dense, unequal length, beige (RAL 1001) to clay brown (RAL 8003), olive brown (RAL 8008), with a serrulate margin. ***Stipe*** 1.5–4 cm long, 1–3 mm thick, cylindrical, when young oyster white (RAL 1013) to light ivory (RAL 1015), with fibrillous to flocculose veil traces, when mature olive brown (RAL 8008) to fawn brown (RAL 8007), surface pruinose, longitudinally fibrillose-striate, base subbulbous.

***Basidiospores*** (60/3/3) (6–)6.5–7.7(–7.9) × 3.7–4.3(–4.4) μm, *Q* = (1.54–)1.58–1.9(–1.97), *Q_m_* = 1.74(±0.09), face view slightly ellipsoid to oblong, side view amygdaliform, occasionally phaseoliform, thick-walled, oil droplets present, germ pore absent, ochre brown (RAL 8001) to clay brown (RAL 8003) in KOH solution. ***Basidia*** 15–28 × 6–9 μm, clavate to nearly cylindrical, 4-spored, sterigmata 2–8 μm long, with vacuolar contents. ***Cheilocystidia*** 20–33 × 7–11(–12) μm, lecythiform, with capitula 3–6 μm wide, lamellae edge sterile. ***Pleurocystidia*** absent. ***Caulocystidia*** 24–45(–48) × 6–12(–15) μm, lecythiform, with capitula 4–6 μm wide, mixed with clavate to nearly spheropedunculate elements. ***Pileipellis*** epithelioid hymeniderm, composed of spheropedunculate cells (21–)30–56(–61) × 16–22(–24) μm, base with nut brown (RAL 8011) pigment. ***Pileocystidia*** absent. All structures have clamp connections. Weakly positive reaction with ammonia, forming diamond-shaped crystals.

##### Habitat.

In summer and autumn, scattered or in groups on the humus layer of broad-leaved forests or on rotten wood sticks.

##### Known distribution.

Jilin Province, China.

##### Additional material examined.

China • Jilin Province, Jilin City, Jiaohe City, Qianjin Forest Farm, 25 August 2023, 43°57'03"N, 127°41'54"E, alt. 444 m, Xia Wang, 3R11 (HMJAU65110). China • Jilin Province, Jilin City, Huadian City, Hongshi National Forest Park, 3 September 2024, 42°55'42"N, 127°06'14"E, alt. 325 m, Tian-yu Zhang, 4K26 (FJAU78874). China • Jilin Province, Changchun City, Lianhua Mountain, 19 September 2024, 42°52'0"N, 125°44'4"E, alt. 245 m, Han-bing Song, S24091921 (FJAU78875). Sequences were obtained from all specimens mentioned above, and their morphology was also examined.

##### Notes.

This species is placed in sect. *Intermediae* ser. *Brunnea* based on its veiled pileus and lecythiform cheilocystidia (Hausknecht and Krisai-Greilhuber 2007). Its main distinguishing features are 4-spored basidia, basidiospores without a germ pore, and the absence of pileocystidia. Other species in sect. *Intermediae* that also lack a germ pore include *P. pseudoampullaceocystis*, *P. sulciceps* T. Bau & H.B. Song, and *P. horchinensis* T. Bau & H.B. Song (Karich 2020; Song and Bau 2024). This species differs from *P. pseudoampullaceocystis*, which has 2-spored basidia, basidiospores 9–11.5 μm long, and cheilocystidia that include both utriform and lageniform elements (Karich 2020). It differs from *P. sulciceps*, which has loosely arranged lamellae and possesses pileocystidia that are lageniform and lecythiform (Song and Bau 2024). It differs from *P. horchinensis*, which has basidiospores that are distinctly reniform to phaseoliform and possesses lecythiform pileocystidia (Song and Bau 2024). Phylogenetically, this species is closely related to *P. micropora* and *P. bispora*, but the three species can be easily distinguished: *P. micropora* has a germ pore less than 1 μm in diameter (some germ pores are indistinct) and lecythiform pileocystidia, whereas *P. bispora* has 2-spored basidia and lecythiform pileocystidia and shows a negative reaction to ammonia (Song and Bau 2024).

### Key to species of *Pholiotina* sect. *Pholiotina* ser. *Teneroides*

**Table:** 

1	Predominantly 2-spored	**2**
–	Predominantly 4-spored	**3**
2	Cheilocystidia with crystals at the apex	***Pholiotina altaica***
–	Cheilocystidia without crystals	***P. teneroides***
3	Coprophilous	**4**
–	Non-coprophilous	**5**
4	On horse dung	***P. stercoraria***
–	On elephant dung	***P. indica***
5	At maturity, the pileus is larger than 3 cm	**6**
–	At maturity, the pileus is smaller than 2.5 cm	**7**
6	Context has a faint farinaceous odor	***P. procera***
–	Context odorless	***P. utricystidiata***
7	Basidia subutriform to cylindrical, with a median constriction	***P. constrictibasidiata***
–	Basidia ventricose to clavate	**8**
8	Stipe length greater than 5 cm, often flexuous	***P. flexipes***
–	Stipe length less than 4.5 cm, not curved	***P. tucumana***

### Key to species of *Pholiotina* sect. *Vestitae*

**Table:** 

1	Basidiospores without germ pore	**2**
–	Basidiospores with a distinct germ pore	**3**
2	Stipe base subbulbous	***Pholiotina serrata***
–	Stipe base hardly swollen	***P. vestita***
3	Cheilocystidia with resinous plaques	***P. resinosocystidiata***
–	Cheilocystidia without resinous plaques	**4**
4	Context with a faint *Pelargonium* odor	**5**
–	Context odorless	**6**
5	Basidiospores average 6.9–7.8 μm in length	***P. velata***
–	Basidiospores average 9.0–11.1 μm in length	***P. nemoralis***
6	Basidia rare	***P. raribasidiata***
–	Basidia common	***P. exannulata***

### Key to species without germ pore of *Pholiotina* sect. *Intermediae*

**Table:** 

1	Predominantly 2-spored	***Pholiotina pseudoampullaceocystis***
–	Predominantly 4-spored	**2**
2	Pileocystidia absent	***P. jilinensis***
–	Pileocystidia present	**3**
3	Pileocystidia mix lageniform and lecythiform	***P. sulciceps***
–	Pileocystidia only lecythiform, rare	***P. horchinensis***

## Discussion

Using the phylogenetic framework of Tóth et al. (2013) and Song (2025), the phylogeny of *Pholiotina* was reconstructed using a concatenated dataset of ITS, 28S, and *tef*1-α sequences. The results show that *Pholiotina* comprises two distinct evolutionary lineages: one containing sect. *Pholiotina* and the other containing sect. *Vestitae* and sect. *Intermediae* (1/100). Integrating morphological evidence, specimens collected from Jilin Province and the Xizang Autonomous Region were examined, and three new species were recognized: *P. constrictibasidiata* from Xizang (sect. *Pholiotina*), *P. raribasidiata* from Jilin (sect. *Vestitae*), and *P. jilinensis* from Jilin (sect. *Intermediae*).

Within sect. *Pholiotina*, most species cluster in a strongly supported monophyletic clade (1/100). The exceptions are *P. hadrocystis* and *P. aporos*, which fall outside that clade on poorly supported branches, leaving their phylogenetic positions unresolved (Fig. 1). Among the three morphological series recognized in the section (ser. *Aporos*, ser. *Vexans*, and ser. *Teneroides*), only ser. *Teneroides* is strongly supported by both morphological and phylogenetic evidence, forming a monophyletic clade sister to *P. vexans* with strong support (1/100) (Hausknecht and Krisai-Greilhuber 2007; Hausknecht 2009). The newly described species *P. constrictibasidiata* also fits within ser. *Teneroides* both morphologically and phylogenetically. This addition brings the number of species in sect. *Pholiotina* to 29 and in ser. *Teneroides* to nine (Hausknecht 2009; Siquier and Salom 2017; Siquier et al. 2021; Musumeci 2023; Burenbaatar et al. 2024; Song and Bau 2024). Sequence data are currently available for approximately 18 species of sect. *Pholiotina*, four of which belong to ser. *Teneroides* (Tóth et al. 2013; Siquier and Salom 2017; Siquier et al. 2021; Musumeci 2023; Burenbaatar et al. 2024; Song and Bau 2024).

Within sect. *Vestitae*, two independent monophyletic clades are currently resolved, making the section paraphyletic: one clade corresponding to ser. *Vestita* (0.99/90) and the other to ser. *Appendiculata* (0.85/85). Among the morphological series recognized in sect. *Vestitae* (ser. *Vestita*, ser. *Resinosocystidiata*, and ser. *Appendiculata*), ser. *Vestita* and ser. *Appendiculata* are supported by both morphological and phylogenetic evidence. In contrast, the monotypic ser. *Resinosocystidiata* still lacks sequence data for its type species, leaving its phylogenetic position unresolved (Hausknecht and Krisai-Greilhuber 2007; Tóth et al. 2013). The newly described species *P. raribasidiata* fits within ser. *Appendiculata* both morphologically and phylogenetically. This addition brings the number of species in sect. *Vestitae* to 10 and in ser. *Appendiculata* to seven (Hausknecht 2009; Liu and Bau 2018). Currently, only five species in sect. *Vestitae* have available sequence data, of which two belong to ser. *Appendiculata* (Tóth et al. 2013; Liu and Bau 2018). Given this limited molecular sampling, resolving the paraphyly of sect. *Vestitae* will require expanding species sampling within the section and obtaining sequences from the remaining five known species.

Within sect. *Intermediae*, all species form a single monophyletic clade (0.99/90), with the exception of *P. pseudoampullaceocystis*, whose phylogenetic position remains uncertain and is only weakly supported (–/83) (Karich 2020). In the phylogenetic tree of Song and Bau (2024), this species was placed in a transitional position between sect. *Vestitae* and sect. *Intermediae*, a result that contrasts with the present study and further indicates that its phylogenetic position is unresolved. In contrast, the two morphological series recognized in the section, ser. *Intermedia* and ser. *Brunnea*, are well supported by both morphological and phylogenetic evidence. The newly described species *P. jilinensis* also fits within ser. *Brunnea* both morphologically and phylogenetically. This addition brings the number of species in sect. *Intermediae* to 16 and in ser. *Brunnea* to 15 (Hausknecht 2009; Karich 2020; Song and Bau 2024). Currently, 15 species in the section have sequence data, 14 of which belong to ser. *Brunnea*, with only *P. caricicola* Singer still lacking molecular sequence data (Singer 1989; Tóth et al. 2013; Karich 2020; Song and Bau 2024).

The three new species described in this study increase the known species diversity of *Pholiotina*, and outstanding taxonomic issues in the genus are re-examined based on morphological and phylogenetic evidence. Fully resolving these issues will require additional specimens and sequence data from other known species to improve branch support in the phylogenetic tree, thereby enabling further revision of the genus.

## Supplementary Material

XML Treatment for Pholiotina constrictibasidiata

XML Treatment for Pholiotina raribasidiata

XML Treatment for Pholiotina jilinensis

## Additional information

### Artificial Intelligence (AI) use

The authors accept full responsibility for the content of the manuscript, including the disclosure of any use of AI.

No AI tools were used in the preparation of this manuscript.

### Author ORCIDs

J.-l. Deng https://orcid.org/0009-0004-5062-3908

H.-b. Song https://orcid.org/0000-0002-7440-5444

### Data availability

All sequences have been deposited in GenBank (https://www.ncbi.nlm.nih.gov), and the nomenclatural novelties (new species names) have been registered in MycoBank (https://www.mycobank.org). The data presented in this study have been deposited in the Zenodo repository, accession number https://doi.org/10.5281/zenodo.20409719.
